# Characterization and genome analysis of a psychrophilic methanotroph representing a ubiquitous *Methylobacter* spp. cluster in boreal lake ecosystems

**DOI:** 10.1038/s43705-022-00172-x

**Published:** 2022-09-19

**Authors:** Ramita Khanongnuch, Rahul Mangayil, Mette Marianne Svenning, Antti Juhani Rissanen

**Affiliations:** 1 Faculty of Engineering and Natural Sciences, Tampere University, P.O. Box 541, 33014 Tampere, Finland; 2 Department of Arctic and Marine Biology, UiT, The Arctic University of Norway, 9037 Tromsø, Norway; 3 Natural Resources Institute Finland, Latokartanonkaari 9, 00790 Helsinki, Finland

**Keywords:** Bacteria, DNA sequencing, Functional genomics, Environmental microbiology, Bacterial physiology

## Abstract

Lakes and ponds are considered as a major natural source of CH_4_ emissions, particularly during the ice-free period in boreal ecosystems. Aerobic methane-oxidizing bacteria (MOB), which utilize CH_4_ using oxygen as an electron acceptor, are one of the dominant microorganisms in the CH_4_-rich water columns. Metagenome-assembled genomes (MAGs) have revealed the genetic potential of MOB from boreal aquatic ecosystems for various microaerobic/anaerobic metabolic functions. However, experimental proof of these functions, i.e., organic acid production via fermentation, by lake MOB is lacking. In addition, psychrophilic (i.e., cold-loving) MOB and their CH_4_-oxidizing process have rarely been investigated. In this study, we isolated, provided a taxonomic description, and analyzed the genome of *Methylobacter* sp. S3L5C, a psychrophilic MOB, from a boreal lake in Finland. Based on phylogenomic comparisons to MAGs, *Methylobacter* sp. S3L5C represented a ubiquitous cluster of *Methylobacter* spp. in boreal aquatic ecosystems. At optimal temperatures (3–12 °C) and pH (6.8–8.3), the specific growth rates (µ) and CH_4_ utilization rate were in the range of 0.018–0.022 h^−1^ and 0.66–1.52 mmol l^−1^ d^−1^, respectively. In batch cultivation, the isolate could produce organic acids, and the concentrations were elevated after replenishing CH_4_ and air into the headspace. Up to 4.1 mM acetate, 0.02 mM malate, and 0.07 mM propionate were observed at the end of the test under optimal operational conditions. The results herein highlight the key role of *Methylobacter* spp. in regulating CH_4_ emissions and their potential to provide CH_4_-derived organic carbon compounds to surrounding heterotrophic microorganisms in cold ecosystems.

## Introduction

Methane (CH_4_) is one of the major natural and anthropogenic greenhouse gases, with a global warming potential of approximately thirty times higher than that of CO_2_ over a 100-year time horizon [1]. Lakes, one of the major natural sources of CH_4_ emissions, have recently gained interest as they account for ∼6–7% of the total natural emissions [2, 3]. In particular, the emissions from boreal and arctic lakes are elevated during the ice-free period than at other times of the year [3–6].

In the lake ecosystems, CH_4_ is generally produced by methanogenic archaea in anoxic sediments/layers and eventually emitted upwards toward the water-atmosphere interface. During CH_4_ traveling along the lake water column, various studies have reported the function of aerobic methane-oxidizing bacteria (MOB) as the key factor in controlling these CH_4_ fluxes at the oxic-anoxic interfaces in boreal and arctic lake ecosystems [7–11]. MOB require oxygen as an electron acceptor to oxidize CH_4_ for biomass formation and CO_2_ generation. During hypoxic (i.e., oxygen-limiting) conditions, MOB may shift the cellular metabolism toward fermentation to stabilize their cellular redox potential, either by generating various extracellular organic acids [12] or using nitrate as the terminal electron acceptor via anaerobic respiration [13]. The metabolism of MOB is of great ecological importance as the produced by-products (e.g., methanol, formaldehyde, and organic acids) can serve as growth substrates for surrounding methylotrophic and heterotrophic microorganisms [14, 15]. Furthermore, MOB interacting with a high heterotroph richness in a coculture system could also enhance CH_4_ oxidation as the cross-feeding mechanism might help to reduce the toxic compounds’ inhibitory effect on MOB [14]. Studies on implementing the produced extracellular by-products as biofuel precursors or as industrial platform chemicals also imply the biotechnological importance of MOB [16–18].

The study of metagenome-assembled genomes (MAG) and experimental observations have revealed that MOB in northern lakes have the genetic potential for denitrification and fermentation, resulting in organic acid production [7, 19]. During our previous study on genomic characteristics of boreal lake water column MOB, an attempt to demonstrate organic acid production by MOB in northern lakes was conducted via experimental incubation of natural lake water samples [7]. However, it was not successful due to the low concentrations of organic acids (results not shown in [7]). This suggests that the study on fermentation by lake MOB requires the cultivation of enrichment or obtaining isolates of lake MOB. Up to now, CH_4_ oxidation metabolism in cold conditions, including psychrophilic conditions (0–20 °C), has rarely been studied using enrichment or isolates of MOB [20, 21].

The study on lake MOB isolates would benefit in understanding their function, environmental distribution, and potential to broaden the biotechnological prospects of MOB. Herein, we focused on isolation, characterization, whole genome assembly, and evaluation of organic acid production of a psychrophilic MOB strain isolated from a boreal lake.

## Materials and methods

### Sampling site

Lake water samples from a small humic and O_2_-stratified lake, Lake Lovojärvi in southern Finland (61° 04’N, 25° 02’E), were used as the isolation source [22]. The sampling was conducted at the hypoxic layer (5.75 m depth, temperature 5.2 °C, pH 6.5, and dissolved O_2_ concentration (DO) < 0.3 mg l^−1^) (Supplementary Fig. S1). Lake water samples were pre-filtered through a 50 µm mesh to remove larger plankton. The filtered water was collected in 250 ml glass bottles containing 20% CH_4_ and 80% air (*v/v*) and stored at 5 °C prior to enrichment.

### Enrichment and isolation

In this study, nitrate mineral salts (NMS) medium modified from DSMZ medium 921 with an initial pH of ∼6.80 (Supplementary Table S1) was used as the liquid growth medium of MOB. The solid medium consisted of the NMS liquid medium supplemented with 1.5% Noble agar (Agar-Agar SERVA powder analytical grade, Germany). For MOB enrichment, 0.5 ml lake water was added into a 25 ml sterile serum bottle containing a 5 ml sterile NMS medium. The bottle was sealed with sterile butyl rubber stoppers and aluminum crimps, and the headspace was replaced with 20% (*v/v*) of CH_4_ prior to incubating in static conditions at 5 °C for 42 days. The enriched culture was sub-cultured thrice in the NMS medium prior to streaking onto the NMS agar medium. Agar plates were incubated in an air-tight chamber containing ∼20% CH_4_ and 80% air (*v/v*) in the headspace and placed at 5 °C for ∼60 days. Colonies were picked under a stereo microscope and re-streaked onto NMS agar medium. Heterotroph contamination was checked by streaking colonies on nutrient-rich agar medium (0.5% tryptone, 0.25% yeast extract, 0.1% glucose and 2% agar). Culture purity was confirmed when one cell type was observed under a light microscope, and growth was absent on both nutrient-rich and NMS media without CH_4_ supplementation. The culture purity was also confirmed by high-throughput full-length 16S rRNA gene amplicon sequencing using the Pacbio Sequel platform (Novogene Co. Ltd., Beijing, China).

### Biochemical characterization

The growth tests at different pH, temperatures, and nitrogen sources were conducted in 27 ml sterile glass tubes containing 5 ml NMS medium and an initial culture of optical density at 600 nm wavelength (OD_600nm_) of 0.02. The tubes were subsequently sealed with sterile butyl rubber stoppers and aluminum crimps. The initial headspace gas composition of 20% CH_4_ and 80% air (*v/v*) was established by replacing 20% (*v/v*) of the air headspace with pure CH_4_. The pH test was performed in triplicates (pH 4.7–8.3) at 5 °C under static conditions. The growth temperature test was conducted in duplicate using a temperature-gradient incubator (Terratec Corporation, Hobart, Australia), set in the range of 0–26 °C (at pH 6.8). In the different nitrogen source tests, NMS and ammonium mineral salts (AMS) media (Supplementary Table S1) were used as nitrate and ammonium sources, respectively, to cultivate the isolate. The test was conducted in duplicate. In addition, the total cellular carbohydrate content during the growth in NMS and AMS media was measured at the end of the test. All tests were incubated at static conditions for ∼19–21 days. CH_4_ and CO_2_ compositions in the headspace and OD_600nm_ were monitored daily during weekdays, while O_2_ in the headspace and organic acid concentrations in the liquid medium were measured at the beginning and end of the test. The growth rates (µ) were obtained from linear regression of the plot between Log_10_ optical density versus incubation time. To test nitrogen (N_2_) fixation, the isolate was incubated in 25 ml serum bottles containing sterile nitrate free-NMS liquid medium. The headspace (*v/v*) was supplemented with (i) 20% CH_4_ + 80% N_2_ and (ii) 20% CH_4_ + 5% O_2_ + 75% N_2_ for anaerobic and microaerobic conditions, respectively. The bottles were sealed with sterile butyl rubber stoppers with aluminum crimps and incubated statically at ∼5 °C for over 30 days.

### DNA extraction and identification

Genomic DNA (gDNA) extraction was performed using GeneJET genomic DNA purification kit (Thermo Fisher Scientific, USA). The gDNA was quantified using a Qubit 3.0 Fluorometer and a dsDNA HS Assay Kit (Thermo Fisher Scientific, USA). PCR and amplicon sequencing (Sanger Sequencing method) of the 16S rRNA gene were performed using the identification service offered by Macrogen (Amsterdam, The Netherlands), using primer pairs 27F (AGAGTTTGATCMTGGCTCAG) and 1492R (TACGGYTACCTTGTTACGACTT) for amplification and primer pairs 785F (GGATTAGATACCCTGGTA) and 907R (CCGTCAATTCMTTTRAGTTT) for sequencing. A 16S rRNA gene-based phylogenetic tree was constructed in Mega X using the maximum-likelihood algorithm (generalized time-reversible model) with 100 bootstraps [23]. Besides reference strains, the tree was supplemented with 16S rRNA gene sequences of previously studied environmental MAGs representing *Methylobacter* spp. of lakes and ponds of boreal, subarctic, and temperate areas [24, 25]. For the dataset on multiple lakes and ponds, we chose the representative MAGs of metagenomic Operational Taxonomic Units [mOTUs, classified by Buck et al. [24] at 95% average nucleotide identity cutoff]. The 16S rRNA genes were extracted from MAGs using barrnap (version 0.9) [26].

### Genome sequencing, assembly, and annotation

Library preparation and sequencing for long reads (PacBio Sequel SMRT Cell 1M v2) and short reads (Illumina NovaSeq 6000 platform) were done by Novogene Co. Ltd. (Beijing, China) as previously described by Rissanen et al. [22]. The genomes were de novo assembled using a hybrid assembly strategy in Unicycler (version 0.4.8) with default parameters and “–mode normal” [27]. The assembled genome was annotated using the NCBI Prokaryotic Genome Annotation Pipeline [28–30]. Key functional genes and metabolic pathways of the annotated genome were also predicted and reconstructed using Kyoto Encyclopedia of Genes and Genomes (KEGG) mapping tools [31] with KofamKOALA (https://www.genome.jp/tools/kofamkoala/; accessed 1 February 2022) [32].

The genome-wide phylogenetic tree was built from protein alignments generated in PhyloPhlAn (version 3.0.58; PhyloPhlAn database including 400 universal marker genes and “-diversity low” - argument) [33] using the maximum-likelihood algorithm (PROTCATLG − model) with 100 bootstrap replicates in RAxML (version 8.2.12) [34]. Similar to 16S rRNA gene-based phylogenetic tree analysis as explained above, this analysis was supplemented with environmental MAGs representing *Methylobacter* spp. from lakes and ponds of boreal, subarctic, and temperate areas [24, 25], as well as from temperate wetland [10]. The MAGs were also functionally annotated using Prokka (version 1.13.3) [35] and KofamKOALA, as explained above [32]. Average nucleotide identities (ANI) of the genome with the reference genomes and MAGs were computed using fastANI (version 1.32) [36], while average amino-acid identity (AAI) was calculated using CompareM (version 0.1.2) (https://github.com/dparks1134/CompareM). Digital DNA-DNA hybridization (dDDH) for genome-based taxonomic classification was calculated using the Type Strain Genome Server (TYGS) online service (https://tygs.dsmz.de/; accessed 28 February 2022) [37]. Furthermore, *pmoA* sequences coding for the beta subunit of particulate methane monooxygenase of the MAGs, genome, and reference genomes were subjected to phylogenetic tree analyses using the neighbor-joining method (Jones-Taylor-Thornton model) with 500 bootstraps in Mega X [23].

### Evaluation of organic acid production by the isolate

The organic acid production potential of the isolate was evaluated in batch experiments performed in 120 ml sterile serum bottles. Prior to sealing with sterile butyl rubber stoppers and aluminum crimps, 15 ml of NMS medium (pH 6.8) was added to the bottles. The initial culture inoculated into the medium and CH_4_ concentration in the headspace were previously described in Section *Biochemical characterization*. The experiment was conducted in six bottles and incubated at 10.0 ± 1.0 °C in static conditions for 20 days. On day 20, the experiment was divided into two sets, including the first set (in triplicates) replenished with 20% CH_4_ + 80% air (*v/v*) in the headspace, whereas another set (in triplicate) was used as a control without replenishment. The incubation was subsequently continued under similar conditions until day 33. In this study, organic acid production, OD_600nm_, pH in the liquid medium, and the gas composition in the headspace were periodically monitored every 2 or 3 days. However, in the control test without the replenishment, the gas composition in the headspace was monitored only at the beginning and end of the experiment. NMS medium without cells and an inoculated culture without CH_4_ and air addition were also used as controls.

### Analytical methods

Cell growth was determined using an Ultrospec 500 pro spectrophotometer (Amersham Biosciences, UK). The medium pH was measured using a pH 330i portable meter (WTW, Germany) equipped with a SlimTrode electrode (Hamilton, Germany). To determine the organic acid composition, the cultures centrifuged (15 min at 2700 × *g*) and filtered through a 0.2 µm membrane (Chromafil^®^ Xtra PET 20/25, Macherey-Nagel, Germany) were analyzed using a Shimadzu high-performance liquid chromatography equipped with Rezex RHM-Monosaccharide H^+^ column (Phenomenex, USA) as described in Okonkwo et al. [38]. Gas composition in the headspace (i.e., CH_4_, CO_2_, and O_2_) was measured using a Shimadzu gas chromatograph GC-2014 equipped with a thermal conductivity detector and a Carboxen-1000 60/80 column (Agilent Technologies, USA) as described in Khanongnuch et al. [16]. To obtain further insight into the distribution of carbon in the cell biomass, the total carbohydrate content was evaluated using the phenol-sulfuric acid method [39]. The standard curve of the test was prepared using known glucose concentrations.

### Statistical analysis

The specific growth rate, CH_4_ utilizing rate, and organic acid concentration from different experimental conditions were statistically compared using one-way analysis of variance (ANOVA) (Minitab16.0, USA) with Tukey’s multiple comparison tests. The significance level was the 95% confidence interval, where a *p value* ≤ 0.05 was considered statistically significant.

## Results

### Isolation, characterization, and classification

The isolate was obtained from a single colony forming on NMS agar media after being incubated statically at ∼5 °C for over 60 days. The isolate colonies were tiny, < 0.1 mm in diameter, cream, round and entire (Supplementary Fig. S2a). The cells were small and non-motile cocci (1.0–1.8 µm in diameter) that reproduced by binary fission (Supplementary Fig. S2b).

**Fig. 1:**
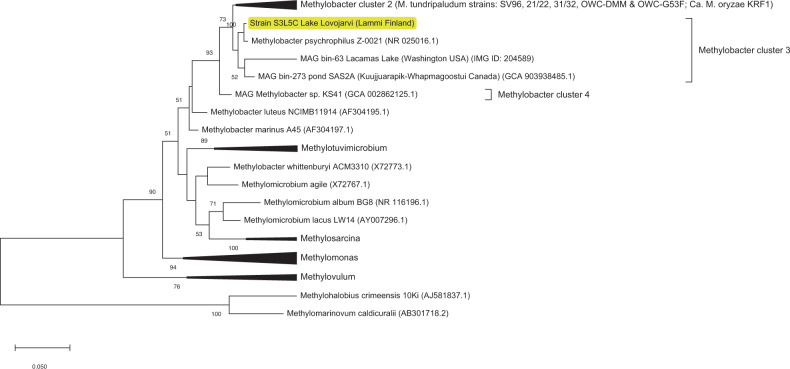
A 16S rRNA gene-based phylogenetic tree of strain S3L5C. The tree shows the position of 16S rRNA gene of strain S3L5C (highlighted in yellow) compared with other pure culture methanotrophic bacteria and metagenome-assembled genomes (MAG). GenBank accession numbers are given in parentheses, and the bar shows a 5% sequence divergence.

For 16S rRNA gene-based identification, the isolate showed 99.5% and 98.9% similarity to *Methylobacter psychrophilus* Z-0021 and *Methylobacter tundripaludum* SV96 isolated from tundra soil [40] and arctic wetland soil [41], respectively (Fig. 1; Supplementary Table S2). The strain formed a separate cluster in the 16S rRNA gene tree with *M. psychrophilus* and MAGs representing MOB from lakes and permafrost thaw ponds (Fig. 1). The isolate was classified in the class *Gammaproteobacteria*, order *Methylococcales*, the family *Methylococcaceae*, and genus *Methylobacter*. Based on the *pmoA* gene-based analysis, the isolate was closely clustered with *M. psychrophilus* and MAGs representing MOB in boreal lake ecosystems (Supplementary Fig. S3).

**Fig. 2:**
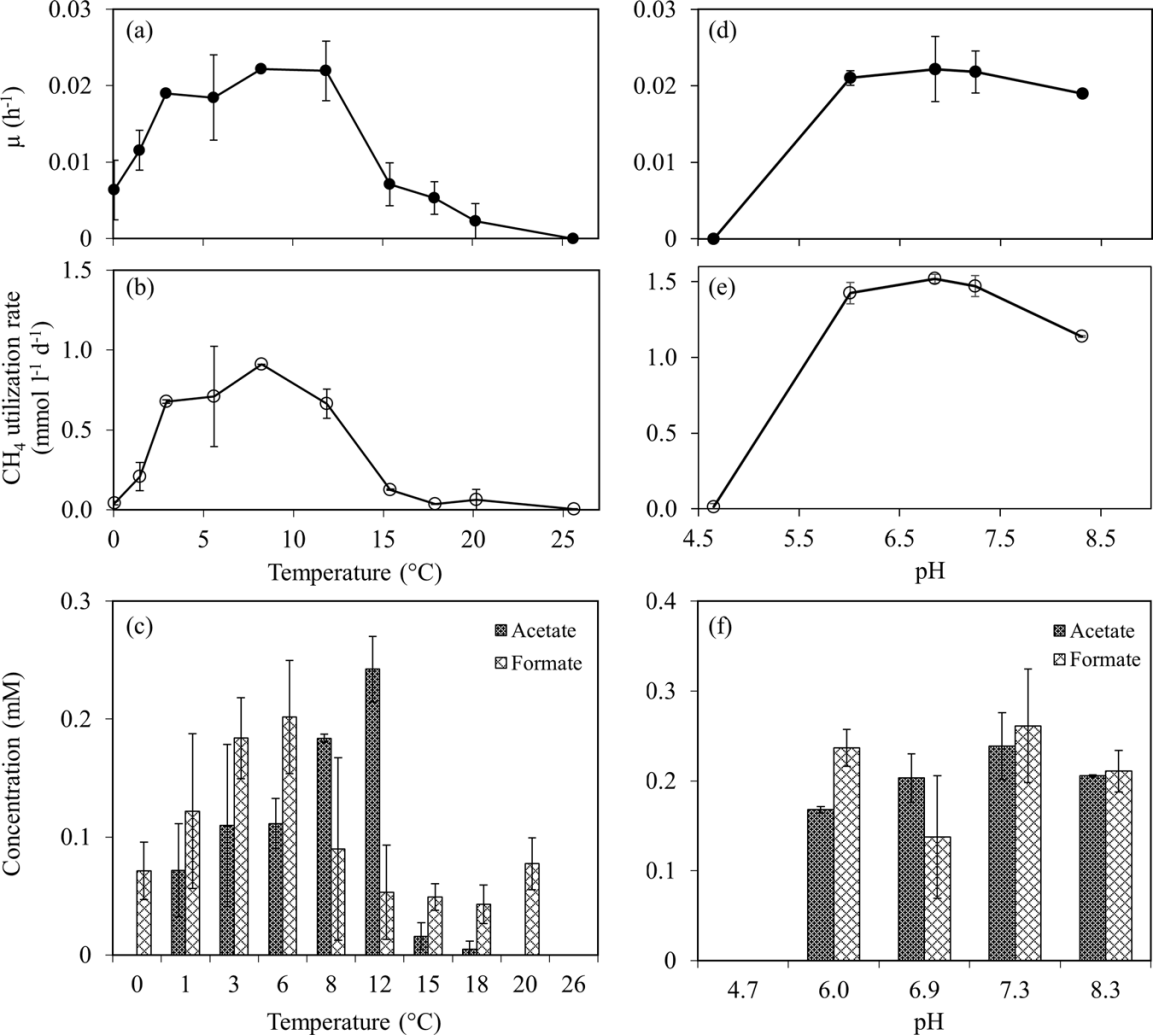
Characterization of strain S3L5C growth at different temperatures and pH. (**a**, **d**) Specific growth, (**b**, **e**) CH_4_ utilization rate, and (**c**, **f**) organic acid concentrations of strain S3L5C at the end of the tests at different temperatures (**a**–**c**) and pH (**d**–**f**). Error bars indicate the standard deviation of duplicates and triplicates for temperature and pH tests, respectively (See Supplementary Table S7 for the dataset).

Table 1 shows the characteristics of *Methylobacter* sp. S3L5C compared to other psychrophilic and psychrotolerant methanotrophic species. During the cultivation, *Methylobacter* sp. S3L5C grew only in the presence of CH_4_ and O_2_. Growth was not observed in the N_2_-fixation test or under anaerobic conditions, but the cells were still viable in those conditions, even after over 1 year of incubation. The strain grew well at 3–12 °C, but the cell growth was not observed above 20 °C, demonstrating its psychrophilic nature (Fig. 2a, b). *Methylobacter* sp. S3L5C could grow well in the pH range of 6.0–8.3, and cell growth was not observed at pH 4.6 (Fig. 2d). At the optimal temperatures (3–12 °C) and pH (6.8–8.3), the specific growth rates (µ) and CH_4_ utilization rate were in the range of 0.018–0.022 h^−1^ and 0.66–1.52 mmol l^−1^ d^−1^, respectively (Fig. 2a, b, d, e). Formate (0.04–0.30 mM) and acetate (0.01–0.24 mM) were identified as the major liquid metabolites in all the tested conditions where the strain growth was present (Fig. 2c, f). The trend of acetate production showed that the increased concentration corresponded with the increase in cell growth (Fig. 2c, f).

**Table 1:** Characteristics of different psychrophilic and psychrotolerant species of methanotrophs.

Strain	*Methylobacter* sp. S3L5C	*Methylobacter psychrophilus* Z-0021	*Methylobacter tundripaludum* SV96	*Methylosphaera hansonii*
Source/habitat	Lake water layer	Tundra soil	Arctic wetland soil	Sediments of marine-salinity Antarctic meromictic lakes
Cell morphology	Cocci	Cocci	Rods	Cocci
Cell size (µm)	1.0–1.8	1.0–1.7	0.8–1.3 × 1.9–2.5	1.5–2.0
Motility	–	–	–	–
Pigmentation	–	–	Pale Pink	*NA*
Growth pH	6.0–8.3	5.9–7.0	5.5–7.9	*NA*
(optimal)	(6.0–7.3)	(6.7)		
Growth temp. (optimal) (°C)	0.1–20 (8–12)	1–20 (10)	5–30 (23)	0–21 (10–13)
Specific conditions for growth	–	–	–	Required seawater
Nitrogen fixation gene (*nif*)	+	*NA*	+	+
G + C content (mol %)	43.3	45–46^a^	47	43–46^a^
Reference	This study	[40]	[41]	[68]

*NA* not available.

^a^G + C content was chemically determined as its genome is not available.

**Fig. 3:**
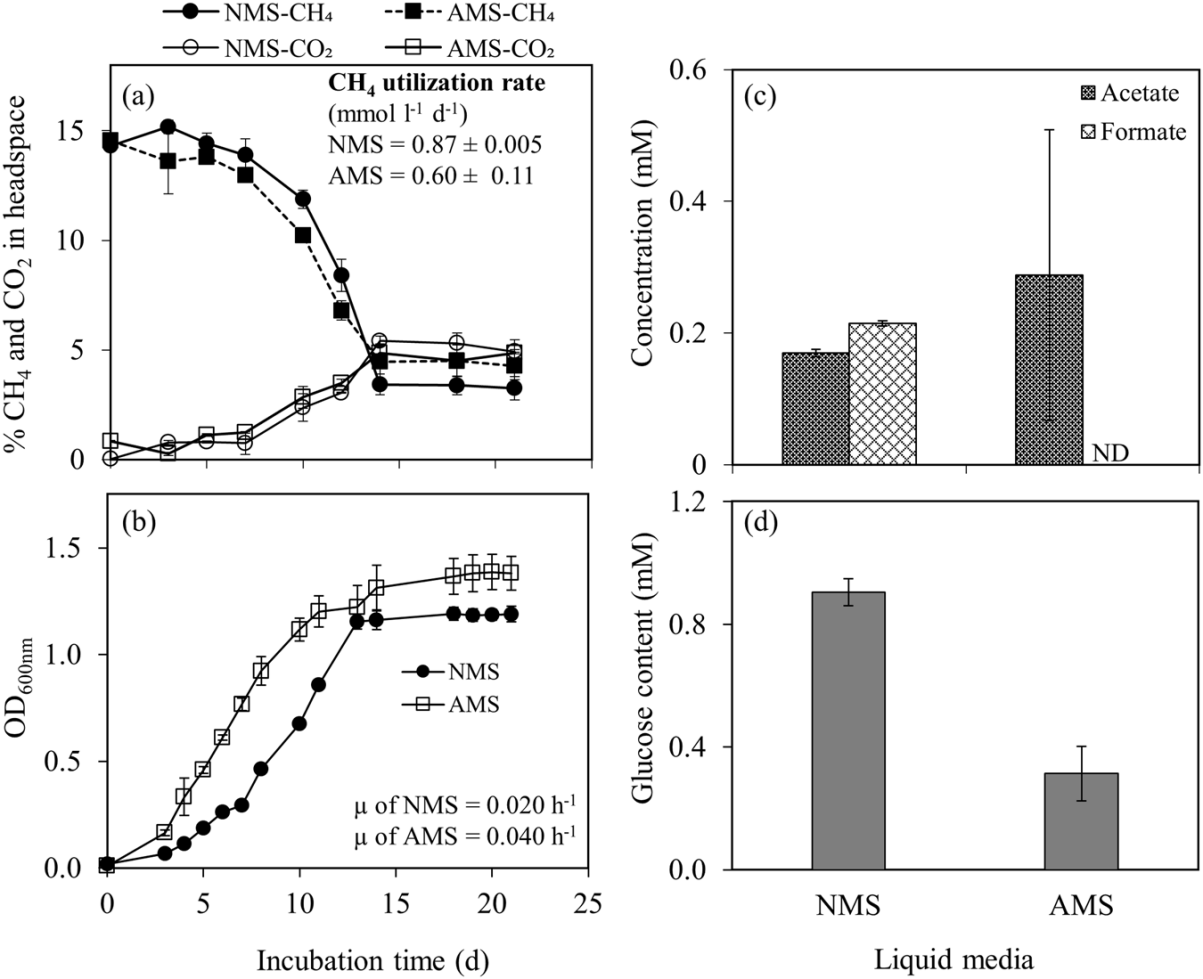
Characterization of strain S3L5C growth with different nitrogen sources. Profiles of (**a**) CH_4_ utilization and CO_2_ production and (**b**) biomass growth during 21-day incubation in nitrate (NMS) and ammonium (AMS) mineral salt media. (**c**) Organic acid production and (**d**) glucose (representing carbohydrate) content of biomass measured at the end of the test (day 21). Error bars indicate the standard deviation of duplicate samples (See Supplementary Table S8 for the dataset).

*Methylobacter* sp. S3L5C could utilize both nitrate and ammonium as the nitrogen source. The specific growth rate in the AMS medium (µ = 0.040 h^−1^) was higher than in the NMS medium (µ = 0.020 h^−1^), whereas the CH_4_ utilization rate was similar in both NMS and AMS media (0.52–0.88 mmol l^−1^ d^−1^) (Fig. 3a, b). In the case of liquid metabolites, acetate and formate were present in the NMS medium; however, only acetate was detected from the cultivations in the AMS medium (Fig. 3c). Furthermore, the total carbohydrate content in biomass cultivated in the NMS medium was 0.91 ± 0.04 mM glucose equivalent which was significantly higher than in the AMS medium (0.31 ± 0.09 mM glucose equivalent) (*p value* < 0.001) (Fig. 3d).

### Genome features of *Methylobacter* sp. S3L5C

**Fig. 4:**
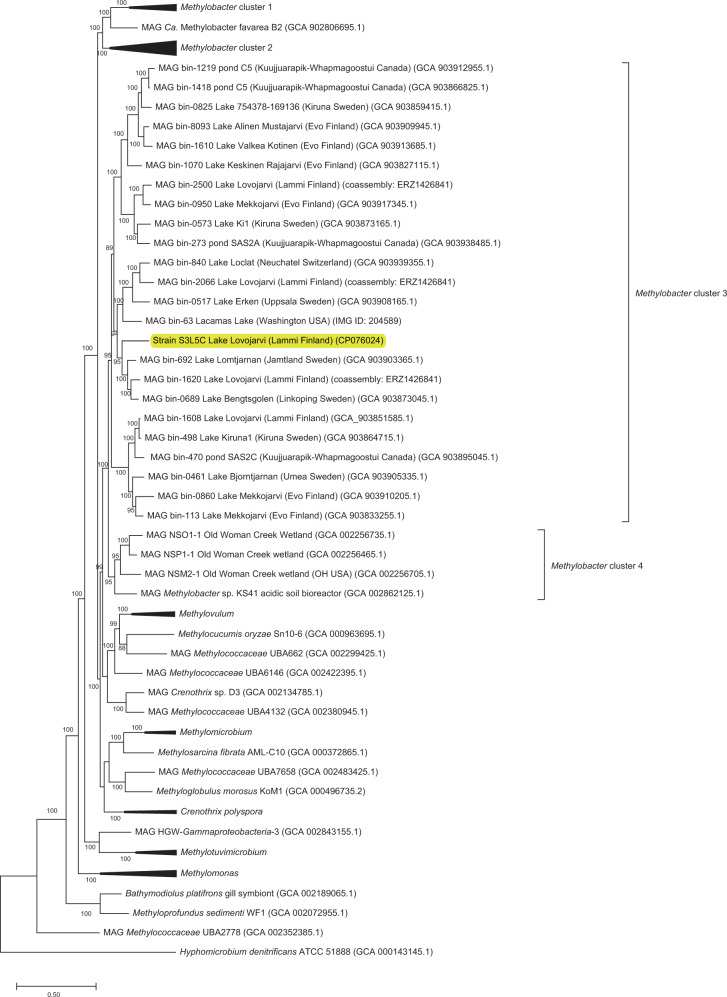
Genome-wide phylogenomic tree of strain S3L5C. The tree was constructed using PhyloPhlAn2 showing the position of strain S3L5C (highlighted in yellow) compared to other cultured methanotrophs and metagenome-assembled genomes (MAGs) of uncultured *Methylobacter* and *Methylococcales* from environmental samples. *Methylobacter* clusters 1 and 2 contain the so far cultured members of *Methylobacter* spp.

*Methylobacter* sp. S3L5C genome contained a single chromosome of 4,815,745 bp (G + C content, 43.3%) consisting of 4176 protein-coding sequences, five copies of rRNA (5S, 16S, 23S rRNA), 50 tRNA genes and 2 CRISPR sequences. *Methylobacter* sp. S3L5C genome was clustered together with MAGs of MOB from lakes and ponds in Finland, Sweden, the USA, Switzerland, and Canada (Fig. 4). Compared to available genomes of other methanotrophic isolates, the S3L5C genome formed a separate species-level branch, and it was not represented by the other isolates (Fig. 4). Regarding similarity indexes for genomic comparison between the SL35C and other MOB, the dDDH was < 25%, while the ANI and AAI values were < 85% (Supplementary Table S3). To recognize the genomic uniqueness of a novel species, same-species delineation thresholds are recommended to be above 70% for dDDH, 95% for ANI, 85% for AAI, and 98.7% similarity with 16S rRNA gene sequences [42–47]. Albeit the high sequence similarity of 16S rRNA genes between *Methylobacter* sp. S3L5C and *M. psychrophilus* Z-0021 (99.5% similarity), we could not confirm whether the strains represented identical species due to the non-existence of genome data of *M. psychrophilus* Z-0021. The latter strain is neither available in the DSMZ repository nor in the All-Russia Collection of Microorganisms (VKM B-2103) culture collection resource (last checked on 24 May 2022), where it was originally deposited [40, 41].

### Predicted metabolic pathways

**Fig. 5:**
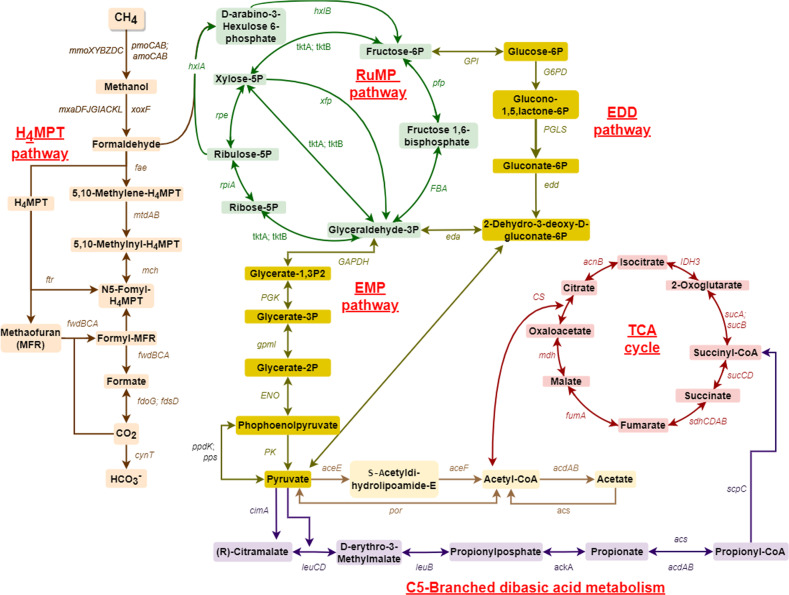
Predicted metabolic pathway of *Methylobacter* sp. S3L5C constructed based on the KEGG mapper [31]. A list of genes is present in Supplementary Table S4. H_4_MPT tetrahydromethanopterin, RuMP ribulose monophosphate, EMP Embden-Meyerhof-Parnas, EDD Enter-Doudoroff, TCA tricarboxylic acid.

The key metabolic pathways in *Methylobacter* sp. S3L5C were predicted based on the KEGG database (Fig. 5). *Methylobacter* sp. S3L5C genome contains all key genes associated with CH_4_ oxidation, including both particulate (*pmoCAB*) and soluble (*mmoXYBZDC*) methane monooxygenases. For the conversion of methanol to formaldehyde, the strain contained both calcium- (*mxaFJGIACKLD*) and lanthanide-dependent (*xoxF*) methanol dehydrogenases. Genes encoding tetrahydromethanopterin (H_4_MPT)-mediated pathway, catalyzing the conversion of formaldehyde into formate, were present in the isolate. *Methylobacter* sp. S3L5C contained a complete set of genes encoding major pathways for formaldehyde assimilation into cell biomass, including ribulose monophosphate (RuMP), Embden-Meyerhof-Parnas (EMP), Enter-Doudoroff (EDD), and phosphoketolases (*xfp*)-based pathways (Fig. 5; Supplementary Table S4). The strain lacked the gene encoding serine-glyoxylate aminotransferase (*sga*), leading to the incomplete serine pathway. Genes present in *Methylobacter* sp. S3L5C also encoded the oxidative tricarboxylic acid (TCA) cycle and C5-branched dibasic acid metabolism.

Gammaproteobacterial MOB can generally oxidize NH_4_^+^ into hydroxylamine (NH_2_OH) using *pmoCAB* [48]. Our experimental observation suggests that the same NH_4_^+^ metabolism might also occur in *Methylobacter* sp. S3L5C. However, the latter did not contain putative *hao* genes encoding hydroxylamine oxidoreductase to convert hydroxylamine to nitrite or nitric oxide [49]. The genome also included nitrate/nitrite conversion genes, i.e., assimilatory nitrate reductase (*nasA*) for reducing nitrate into nitrite and nitrite reductase (*nirBD* and *nirS*) for reducing nitrite into ammonia and nitric oxide. Genes encoding nitrogen fixation (*nifHDK*) were found, although the CH_4_ oxidation and growth were not observed in the culture-dependent experiments of nitrogen fixation under anaerobic and microaerobic (5% O_2_) conditions.

Genes encoding the key enzymes involved in fermentative metabolisms were observed in the genome, including acetyl-CoA synthetase (*acdAB*) catalyzing the conversion of acetyl/propionyl-CoA into acetate/propionate and acetate kinase (*ackA*) for propionate generation from propionyl phosphate. However, some key genes involving acetate synthesis pathways, such as phosphate acetyltransferase (*pta*) and pyruvate dehydrogenase (*poxB*), were not present in the genome. The genome also included malate dehydrogenase (*mdh*) encoding the reversible conversion of oxaloacetate, succinate dehydrogenase (*sdhCDAB*) coupling with succinate production, and NAD^+^-reducing hydrogenase (*hoxFGYH*) encoding H_2_ production. Based on further analyses of environmental MAGs, the similar genetic potential for fermentation and organic acid production is widely dispersed in closely related *Methylobacter* spp. of lakes, ponds, and wetlands of temperate and boreal areas (Supplementary Table S5).

### Organic acid production potential

**Fig. 6:**
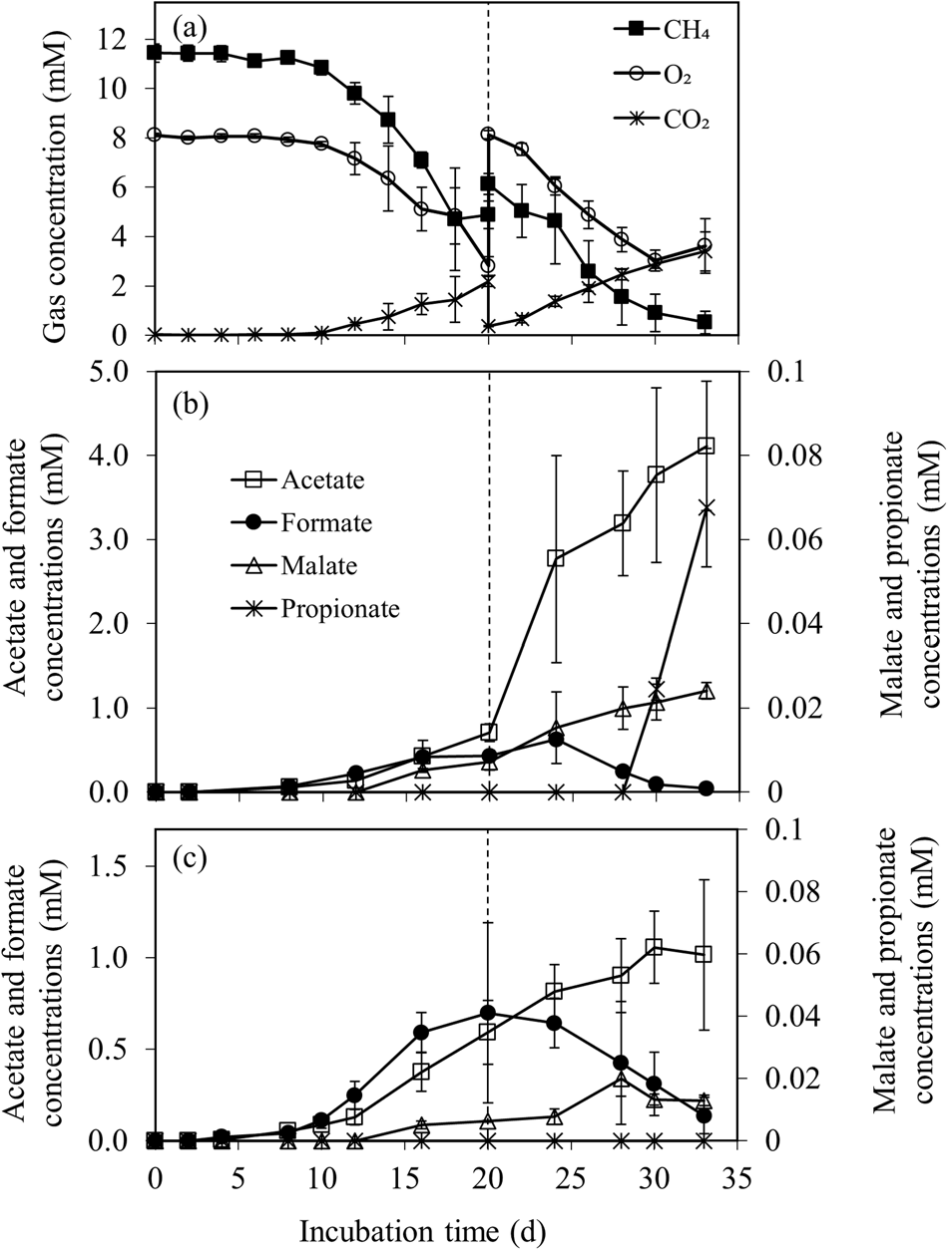
Potential for organic acid production of *Methylobacter* sp. S3L5C. The profiles of (**a**) gas composition in the headspace of the test with 20% CH_4_ and 80% air replenishment on day 20 and concentrations of organic acids in the liquid medium during 33-day cultivation (**b**) with and (**c**) without the replenishment. Error bars indicate the standard deviation of triplicate samples (See Supplementary Table S9 for the dataset).

Batch cultivations were performed to evaluate the strain’s potential to produce organic acids by replenishing CH_4_ and air during incubation (on day 20, Fig. 6a). In the CH_4_ + air replenishment test, the average utilized oxygen-to-methane (O_2_/CH_4_) molar ratio was 1.0 ± 0.5 mol mol^−1^ during cultivation from days 4 to 33 (Supplementary Fig. S4). At the end of the test (day 33), the culture with the replenished headspace had utilized CH_4_ and O_2_ concentrations at ∼1.5–2 times higher than those of the control cultivations without the replenishment, corresponding with the higher CH_4_ and O_2_ concentrations fed into the system (Supplementary Fig. S5a). On day 33, the CH_4_ + air replenishment also increased the biomass growth (up to OD_600nm_ 5.0 ± 0.2), while an OD_600nm_ of 3.2 ± 0.4 was obtained in the control cultivations without CH_4_ + air replenishment (Supplementary Fig. S5b).

Acetate, formate, and malate were observed as soluble metabolites from *Methylobacter* sp. S3L5C cultivations with and without the replenishment, while propionate was only present in the test with CH_4_ + air replenishment (Fig. 6b). Organic acid concentrations gradually accumulated during the cultivation period, except for formate, which was mostly depleted in both experimental conditions (Fig. 6b, c). In the tests with CH_4_ + air replenishment, the accumulated concentrations of acetate (4.1 mM), malate (0.02 mM) and propionate (0.07 mM) were significantly higher than those in the control (*p value* < 0.05) (Fig. 6b, c). In this study, *Methylobacter* sp. S3L5C is hypothesized to convert carbon from CH_4_ (C-CH_4_) into organic acids, CO_2,_ and biomass. The carbon conversion into total accumulated organic acids in the test with CH_4_ + air replenishment (2.5% of the consumed C-CH_4_) was significantly higher than those in the control test (1.2% of the consumed C-CH_4_) (*p value* = 0.037) (Supplementary Table S6). In both experimental conditions, the carbon yields of C-CO_2_ and C-biomass derived from C-CH_4_ were similar in the range of 42.2–46.3% and 51.1–56.4%, respectively (*p value* = 0.124) (Supplementary Table S6).

## Discussion

Strain S3L5C, isolated and classified in this study as *Methylobacter* sp., has been previously reported to be a dominant genus in the oxic-anoxic transition zone in boreal and subarctic lakes, ponds, and wetlands [7, 8, 10, 11, 50–52]. Our phylogenetic and phylogenomic tree analyses showed that the isolate represents a ubiquitous cluster of *Methylobacter* spp. in lake and pond ecosystems. The *Methylobacter* sp. S3L5C genome contained genes encoding key enzymes involved in aerobic CH_4_ oxidation and organic acid production (fermentative metabolism). According to previous studies and MAG analyses of this study, the genetic potential of *Methylobacter* spp. for organic acid production is widely dispersed in CH_4_-rich aquatic systems (Supplementary Table S5) [7, 10, 25]. Whether or not *Methylobacter* sp. S3L5C is a new species of *Methylobacter*, or a type strain of the previously described *Methylobacter psychrophilus* Z-0021 could not be validated as the strain Z-0021, and its genome is not available. Nevertheless, *Methylobacter* sp. S3L5C represents psychrophilic methanotrophs that rarely exist as isolates.

Organic acid production in MOB is an energy conservation mechanism where pyruvate is oxidized to fermentative by-products instead of biomass formation during substrate-limiting conditions (e.g., CH_4_ and O_2_) [12, 53]. In our study, the average O_2_/CH_4_ uptake ratio during incubation from days 4 to 33 (O_2_/CH_4_ uptake ratio of 1.0 ± 0.5; Supplementary Fig. S4) was lower than the stoichiometric ratio in aerobic CH_4_ oxidation (Eq. 1).

1 $${{{{{{{\mathrm{CH}}}}}}}}_4 + 2{{{{{{{\mathrm{O}}}}}}}}_2 \to {{{{{{{\mathrm{CO}}}}}}}}_2 + 2{{{{{{{\mathrm{H}}}}}}}}_2{{{{{{{\mathrm{O}}}}}}}}\quad \quad - 818\,{{{{{{{\mathrm{kJ/reaction}}}}}}}}$$

These conditions probably induced CH_4_ oxidation with O_2_ limited amount in the system and initiated the accumulation of acetate, propionate, and malate. The O_2_/CH_4_ uptake behavior was similar to the previous studies conducted using *Methylomicrobium buryatense* 5GB1C cultivated under O_2_ and CH_4_ limitation conditions (O_2_/CH_4_ uptake ratio of 1.1–1.6) [54, 55].

When *Methylobacter* sp. S3L5C grew in batch cultivation, acetate was observed as the dominant metabolite, and its concentration was remarkably elevated under the conditions with CH_4_ + air replenishment in the headspace. *Methylobacter* sp. S3L5C genome lacks the genes encoding phosphate acetyltransferase (*pta*) and pyruvate dehydrogenase (*poxB*). Nevertheless, experimental validation on the metabolite production suggests that the acetate synthesis route might be catalyzed by acetyl-CoA synthetase and ligase from acetyl-CoA found in the genome (*acs* and *acdAB*; Fig. 5 and Supplementary Table S4) [56]. To the best of our knowledge, this is the first study to report the capacity of a MOB isolate to produce propionate, a liquid metabolite commonly produced by archaea and facultative anaerobic bacteria under anaerobic/microaerobic conditions [57–59]. Genes encoding propionate production (*acdAB*, *acs*, and *ackA*) were found in the annotated genome of *Methylobacter* sp. S3L5C. Thus, based on the in silico data (Fig. 5; Supplementary Table S4), we hypothesize that the propionate production from *Methylobacter* sp. S3L5C may occur via succinate and citramalate pathways similar to other propionate-producing microorganisms [58]. Formate accumulation in the liquid culture has been observed during unbalanced growth conditions (e.g., under O_2_ limitation and cultivated in methanol as a carbon source) [12, 54, 60]. However, in our study, formate was observed as an intermediate metabolite which was eventually depleted during the cultivation period (Fig. 6b, c). Although the genes encoding succinate dehydrogenase were annotated in the genome, succinate was not detected in any studied conditions.

While some MOB are capable of nitrogen fixation, ammonium and nitrate are widely used as nitrogen sources for MOB biomass assimilation [18, 61]. Our results revealed the effect of different nitrogen sources (i.e., nitrate and ammonium) on *Methylobacter* sp. S3L5C growth. The strain favored ammonium for biomass assimilation, resulting in a significantly higher growth rate than the nitrate medium (Fig. 3b). The efficient growth in the ammonium medium can be attributed to the original lake environment (high ammonium content, DO < 0.3 mg l^−1^, Supplementary Fig. S1) from which *Methylobacter* sp. S3L5C was isolated. In the previous study on the bacterial community in anoxic brackish groundwater enrichments, *Methylobacter* sp. was observed as a predominant methanotroph in the CH_4_ and ammonium-rich medium, but it was absent in the nitrate-supplemented medium [62]. However, studies on different gamma- and alphaproteobacterial MOB have reported varying responses to the use of nitrate or ammonium as the optimum nitrogen source [13, 60]. For instance, Tays et al. [60] reported that a medium containing ammonium was optimal for the growth of *Methylocystis* sp. Rockwell, albeit with lower lipid content (fatty acid methyl ester, FAME) compared to the growth in nitrate medium [60]. In some circumstances, nitrate favored the growth of *Methylomonas denitrificans* FJG1 under O_2_-limited conditions [13]. In our study, the strain’s biomass collected from the ammonium medium had significantly less carbohydrate (sugar) content than the cells growing on the nitrate medium (Fig. 3d). This result suggests that the use of different nitrogen sources can be adopted as a strategy to target the major biomass composition (protein, lipid, and carbohydrate contents) during *Methylobacter* sp. S3L5C cultivation. In methanotrophic biomass, carbohydrate commonly represents a carbon sink which might not be preferable for biotechnological applications (e.g., single cell protein and microbial lipid-derived fuels) [55, 63, 64].

In conclusion, our results on *Methylobacter* sp. S3L5C and comparative MAG analyses suggest that *Methylobacter* spp. play a key role in mitigating atmospheric CH_4_ emissions and synthesizing organic acids in boreal and subarctic aquatic ecosystems. The findings suggest that the organic acids produced by *Methylobacter* spp. could serve as a carbon source for surrounding heterotrophic microorganisms to sustain a functioning ecosystem [14, 65]. Furthermore, *Methylobacter* sp. S3L5C may provide an alternative source of CH_4_-derived metabolites for biotechnological applications, especially in cold systems. For example, organic acid-rich spent media can be used to cultivate recombinant heterotrophs to generate value-added compounds [16, 66, 67]. However, further studies on the effect of alternative electron acceptors (e.g., nitrogen oxides) on metabolism and organic acid production of *Methylobacter* sp. S3L5C and process optimizations are required to enhance the production of CH_4_-derived products.

## Supplementary Information

Supplementary Information

Supplementary Tables

## Data Availability

The 16S rRNA gene sequence of *Methylobacter* sp. S3L5C is deposited at GenBank under accession number OM479427. The draft genome assembly of *Methylobacter* sp. S3L5C is available at GenBank under accession number CP076024. The raw reads of the genome sequence are deposited in Sequence Read Archive (SRA) data under accession SRR14663858 for short reads and SRR14663859 for long reads. All data generated and analyzed during this study are included in this published paper and its  supplementary information files.
