# Supplementary material for: Characterization and genome analysis of a psychrophilic methanotroph representing a ubiquitous Methylobacter spp. cluster in boreal lake ecosystems
Source: ISME Commun. 2022 Sep 19;2:85. doi: 10.1038/s43705-022-00172-x (PMC9723741; doi:10.1038/s43705-022-00172-x)
Supplement: Supplementary file 1 — Supplementary Information [file 43705_2022_172_MOESM1_ESM.pdf]

Supplementary Information includes two files

1. Supplementary Figures (.pdf) (included in this document)

2. Supplementary Tables (.xlxs)

Supplementary Figures

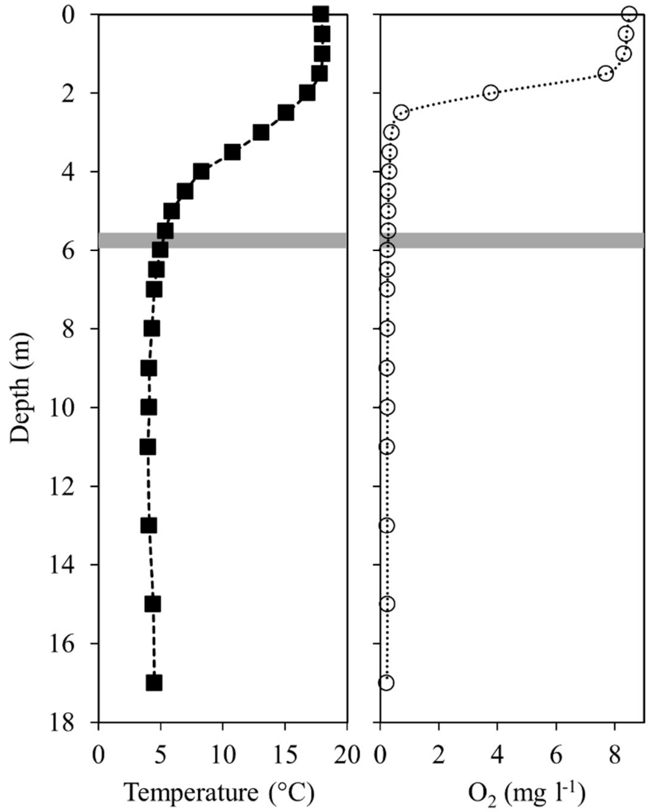

	Sampling point 1	Sampling point 2
Depth (m)	3.25	5.75
N/NH_4^+ ($\mu g\ l^{-1}$)	10	110
P/PO_4^{2-} ($\mu g\ l^{-1}$)	4	32
$N/NO_2^- + NO_3^-$ ($\mu g\ l^{-1}$)	14	8
pH	6.83	6.5
Temperature (°C)	10.8-13.1	5.0-5.4
O_2 ($mg\ l^{-1}$)	0.34-0.40	0.26-0.28†

Fig. S1. Water column depth profiles of temperature and dissolved oxygen (DO) of Lake Lovojärvi and physicochemical characteristics of sampling points (on September 3, 2019). Gray highlights represent sampling points of lake water used for MOB enrichment. † The O_2-measuring instrument (ProODO) has its lowest detection limit at around 0.3 mg l^{-1}. Thus, the actual O_2 concentration is not known.

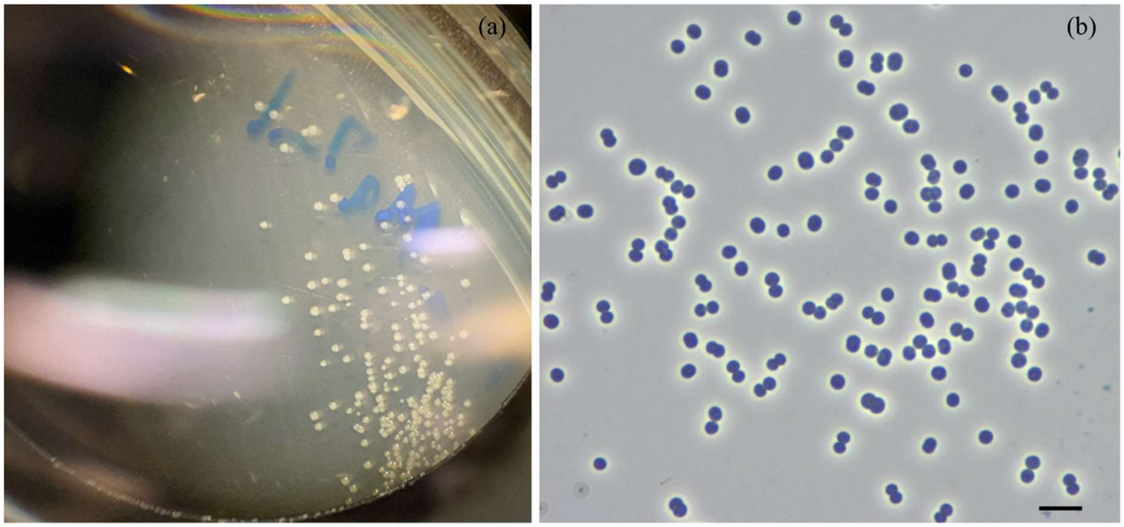

Fig. S2. (a) Photograph of *Methylobacter* sp. S3L5C colonies with a size of < 0.1 mm diameter under a stereo microscope. (b) Phase-contrast light micrograph of cells of the strain S3L5C; bar, 5 µm.

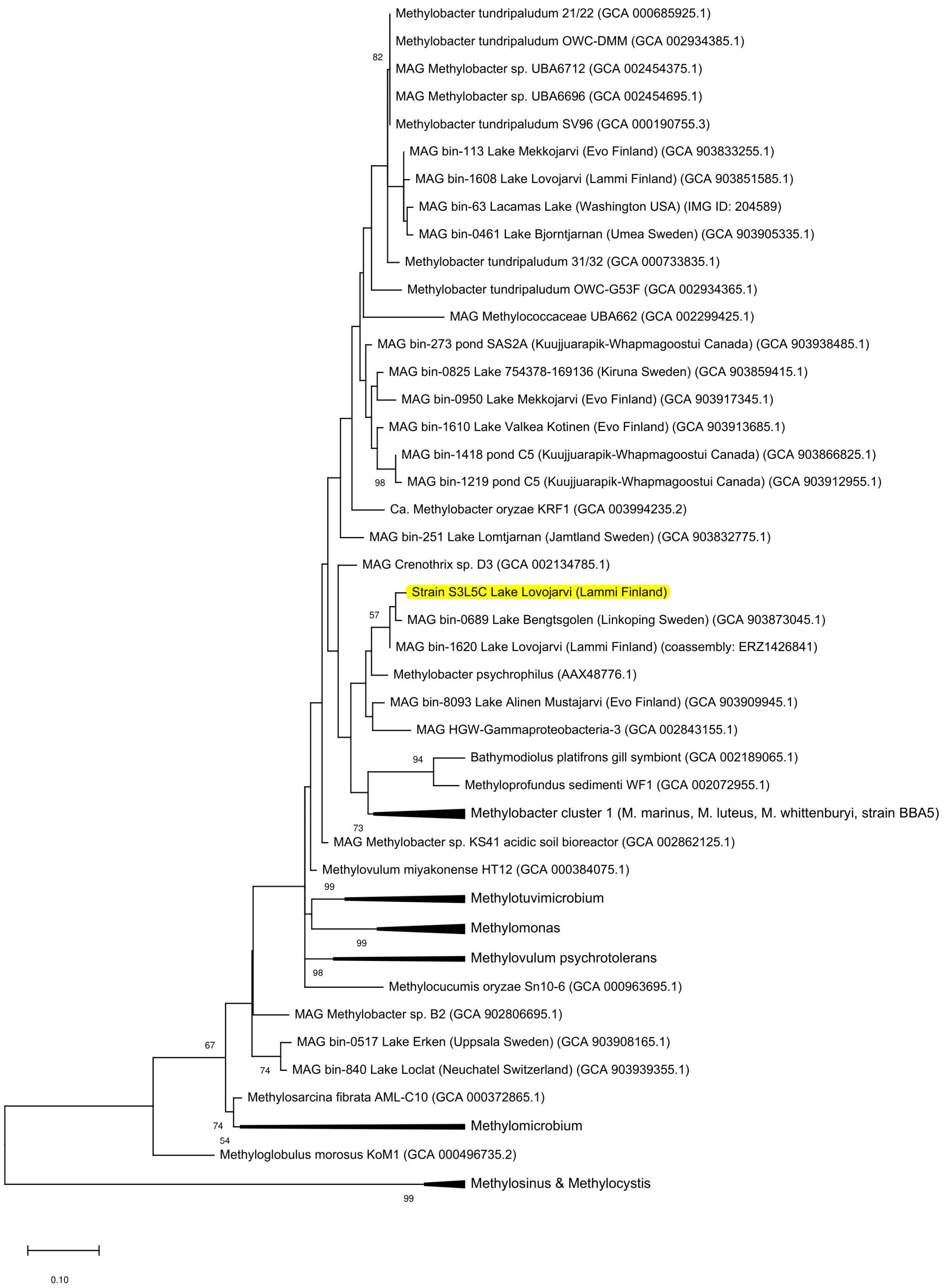

Fig. S3. Phylogenetic tree based on pmoA beta subunit genes of *Methylobacter* sp. S3L5C (highlighted in yellow) compared with other pure culture methanotrophic bacteria and metagenome-assembled genomes (MAG). GenBank accession numbers are given in parentheses, and the bar shows 10% sequence divergence.

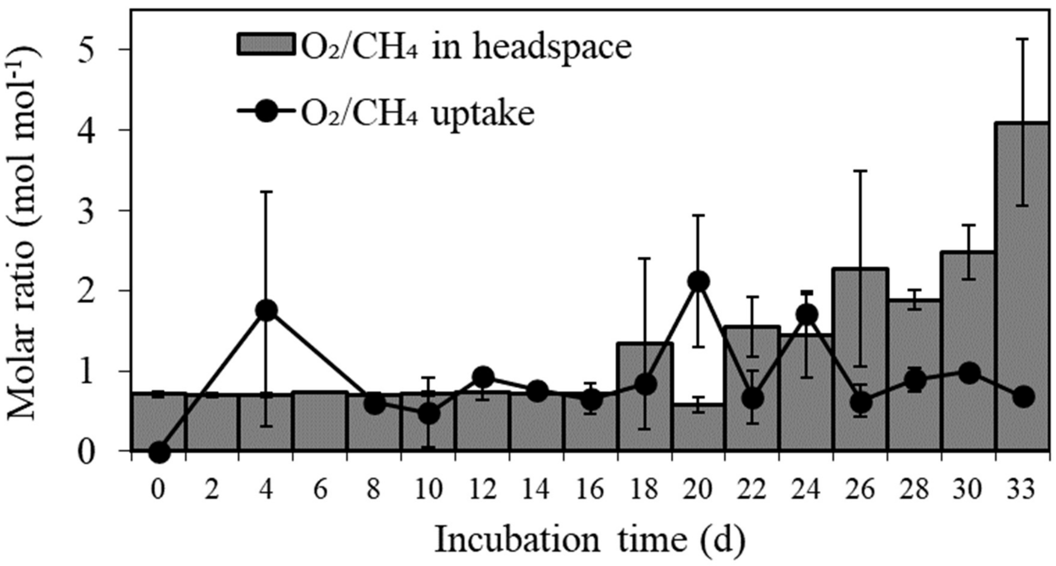

Fig. S4. Profiles of oxygen-to-methane (O_2/CH_4) molar ratio in headspace and uptake during 33-day incubation in the batch tests with 20% CH_4 and 80% air replenishment on day 20. Error bars indicate the standard deviation of triplicate samples (See Supplemental Table S10 for dataset).

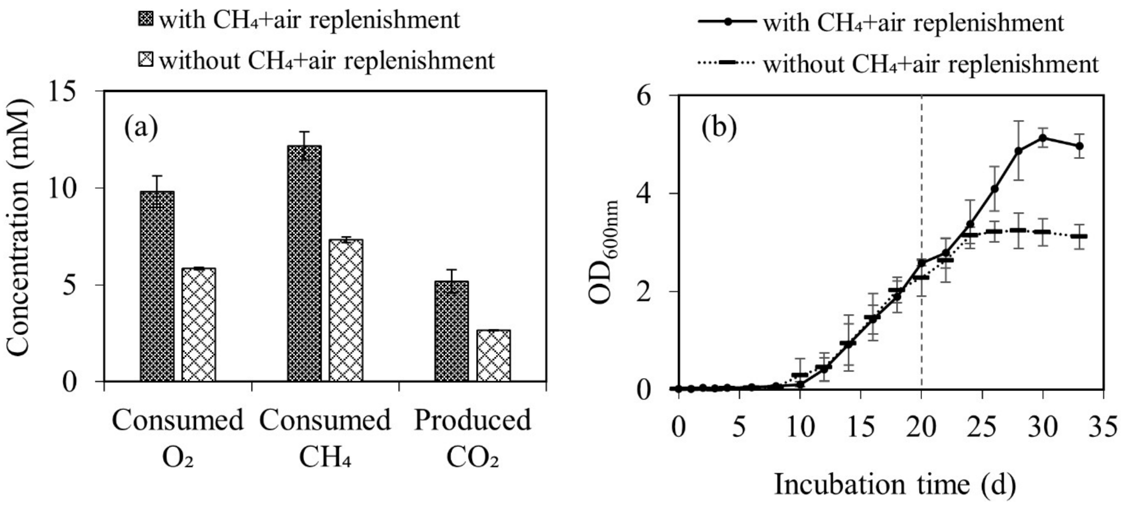

Fig. S5. (a) The consumed O_2 and CH_4 and produced CO_2 at the end of the test (day 33) and (b) biomass growth of *Methylobacter* sp. S3L5C during 33-day incubation between the tests with 20% CH_4 and 80% air replenishment on day 20 and the control without the replenishment.
